# Osteoporosis

**Published:** 1898-10

**Authors:** George H. Berns

**Affiliations:** Brooklyn, N. Y.


### OSTEOPOROSIS.

By George H. Berns, V.S.,
BROOKLYN, N. Y.

In 1890, before the Long Island Veterinary Society, I had the privilege of reading a short paper on "Osteoporosis," which was published in the *American Veterinary Review and Journal of Comparative Medicine and Surgery* at that time. In that paper I briefly considered the history, symptoms presented, differential diagnosis, usual unfavorable terminations, pathological anatomy as described by Williams, Varnell, and others, of England, location of stables and conditions under which most frequently found, and concluded by venturing the following theory as to its probable causes:

Considering the cases that have come under my observation, and more particularly the conditions and location of the stables in which these cases were found, I cannot help but conclude that a specific germ, vegetable or animal, or perhaps a gas, the development or generation of which is favored by certain soils and certain conditions, is the most probable cause, and that this substance, whatever it may be, finding its way into the bodies of animals, acts specifically upon the osseous system, and causes degeneration of a destructive character.

From our present knowledge it would be folly to attempt treatment. Prevention, however, should be aimed at; and during the last six months I have made an effort to have all animals showing the slightest suspicious symptoms of osteoporosis removed to other stables, and in all stables where the disease appeared repeatedly I have had the floors taken up, and two or three feet of urine-and-manure-saturated soil removed, the old flooring-boards and sleepers carefully cleaned and disinfected, and a new supply of good fresh sandy soil put down before the floors were replaced. In cases where it was possible to have the floors raised twelve or eighteen

[1] Read before the New York State Veterinary Society, September, 1898.

inches from the ground, I have strongly advocated to owners the advisability of doing so. As these preventive experiments have been tried by me for the limited period of six months only, I am not prepared to say at this time that they are of any special benefit.

During the last eight years a very large number of cases have come under my observation, and the opinion expressed as to the infectious or possibly contagious character of the disease, I think, has been to a large extent confirmed.

As veterinary examiner to the Brooklyn Retail Grocers' Association for over fifteen years, I have had a most excellent opportunity to study this disease among coarsely-bred, small draught-horses kept under conditions seldom met with in any other line of practice. These horses, as a rule, are bought at the age of six, well fed and cared for, but in most cases kept in small, one-story brick or frame structures, without any sewer connections or proper ventilation, with manure-holes frequently in a corner of the stable, and generally situated in the back-yard of a corner grocery store.

The Brooklyn Retail Grocers' Association is an incorporated society with a membership of over one thousand; each member owns at least one horse, and most all of them have their horses protected by insurance in their society.

Brooklyn grocers, as a class, do not believe in swapping horses; therefore, when a young animal finds its way into a grocer's stable, the chances are that he will remain there as long as he is fit for work, and will be penned up in a small single stall, with hardly room to lie down, and compelled to inhale the close atmosphere contaminated with foul-smelling gases and no doubt millions of microscopical organisms generated by decayed wooden floors, and the damp urine-and-manure-saturated soil under the floors from eighteen to twenty hours each day.

Among these horses I have found osteoporosis extremely prevalent, and out of a total mortality according to the records of the society of from 4.5 to 5 per cent. per annum, 1.5 per cent. are caused by this disease alone, the victims either dying from exhaustion when down and unable to rise, or being destroyed as unfit for further use. I believe I am safe in saying that from 5 to 6 per cent. of all the grocers' horses in Brooklyn, kept under conditions described above, are suffering from osteoporosis in more or less advanced stages. I know of several one-horse stables of this kind in which two or three horses, which were examined by me when purchased and found in good general health, developed this disease successively within a year or two.

Now, let us for a moment consider the existence of this disease among horses kept in large, airy, well-ventilated and properly-drained stables, which are obliged to spend from eight to twelve hours a day in harness, and are therefore out in the open air.

Sixteen years' experience as veterinarian to the stables of A. & S., a large dry-good house in Brooklyn, keeping over 125 head of ordinary common-bred delivery horses, has not revealed one case of osteoporosis. During my ten year's connection with L. & S., owning a stable of 150 heavy brewers' horses; fifteen years' connection with O. & L., stables of about 60 head of brewers' horses, and nineteen years' connection with J. H. B. Co.'s stables of about 90 head of brewers' horses, not a single case of osteoporosis presented itself in either of these stables, and I could enumerate twenty-five or thirty more first-class stables in Brooklyn in which from five to twenty-five horses are kept, and no osteoporosis has ever developed.

During the last seven or eight years, in all cases of osteoporosis which developed in small and badly-drained stables, I have had the floors ripped up, the soil under floors removed to the depth of several feet, new clean soil or coal-cinders substituted, new sleepers and floors put down, stables ventilated, manure-pits closed up and placed outside of buildings, etc., with extremely gratifying results. In stables so renovated, in which two or three horses had developed big head successively in three or four years, no more cases have appeared since these simple sanitary measures were established.

Again, I have never seen a case of osteoporosis recover if it was kept in the same stable in which it had developed the disease; but send the animal away to pasture or to another stable in a distant locality, and there is, according to my experience during the last eight years, at least a chance for a good and serviceable recovery.

Some of our best authorities and most careful observers and investigators, as Friedburger and Fröhner, of Germany, W. L. Williams and Faville, of this country, and several others, regard osteoporosis as identical with rhachitis and osteomalacia in its pathological anatomy, and perhaps justly so; but, as both of these conditions are supposed to be caused by defective assimilation or a lack of food containing lime in sufficient quantity, I cannot understand how a bone which has been fully and regularly developed in a colt, remaining in a perfectly healthy condition after the animal is fully matured for a number of years, should become enlarged to two or three times its normal size, as is frequently the case of the rhami of the lower jaw and facial bones in osteoporosis by simply a want of earthy material in the system. I should think that a deficiency of these constituents would rather tend to produce a diminution in size and, as is the case in osteomalacia in adults, softening of the bones. It seems to me that it requires something that acts as an irritant specifically upon the osseous system, causing metastatic inflammation and the peculiar enlargement and destructive degeneration which are always present; and until this matter is thoroughly cleared up by some of our enthusiastic, painstaking, and highly esteemed investigators, I shall continue to remove my patients to other localities, if possible, and maintain the strictest sanitary precautions.

